# Spatial–Temporal Analysis of Value Network Approach Application in Food Production Sciences

**DOI:** 10.3390/foods15071168

**Published:** 2026-03-31

**Authors:** Javier E. Vera-López, Alberto Santillán-Fernández, Arely del R. Ireta-Paredes, Iban Vázquez-González, Ramiro Reyes-Castro, Alfredo E. Tadeo-Noble, Jaime Bautista-Ortega, Jesús Arreola-Enriquez

**Affiliations:** 1 Bioprospección y Sustentabilidad Agrícola en el Trópico, Colegio de Postgraduados Campus Campeche, Champotón 24450, Campeche, Mexico; jvera@colpos.mx (J.E.V.-L.); reyes.ramiro@colpos.mx (R.R.-C.); jbautista@colpos.mx (J.B.-O.); 2 Programa de Doctorado en Agricultura y Medioambiente para el Desarrollo, Universidad de Santiago de Compostela, 27002 Lugo, Galicia, Spain; 3 Secretaría de Ciencia, Humanidades, Tecnología e Innovación, Colegio de Postgraduados Campus Campeche, Champotón 24450, Campeche, Mexico; tadeo.esteban@colpos.mx; 4 Academia de Administración, Comercio Internacional y Logística, Universidad Politécnica de Texcoco, Texcoco 56250, State of Mexico, Mexico; arely.ireta@uptex.edu.mx; 5 Research Group (GI-1899 ECOAGRASOC), Department of Applied Economics, Escola Politécnica Superior de Enxeñaría, Universidade de Santiago de Compostela, 27002 Lugo, Galicia, Spain; iban.vazquez.gonzalez@usc.es

**Keywords:** agroindustry, content analysis, bibliometrics, non-timber forest products, Universidad Autónoma Chapingo, VOSviewer

## Abstract

Despite the growing number of publications using the value network approach to analyze agro-industrial competitiveness, knowledge gaps persist in other food production sectors. The objective of this study is to analyze, through bibliometric techniques, the scientific articles that have studied the competitiveness of food products using the value network framework. The study will determine the spatial and temporal distribution of the identified food products and detect opportunities for generating new research. Articles from major publishing databases (Elsevier, Scopus, Frontiers, MDPI, and Springer) were considered. The keywords used were “red de valor” and “value network”, combined with “sustainable agricultural production” and “food security”. This information formed the basis of a spatial–temporal analysis and bibliometric indicators using descriptive statistics, as well as keyword and author networks generated with VOSviewer software. A total of 147 scientific articles were documented. The highest growth in publications occurred from 2017 to 2024 and was concentrated in Latin America, Europe, and Asia. Studies in these regions analyzed basic food products such as maize, mango, rice, and coffee in Latin America; wine and bovine milk in Europe; and rice and sugar in Asia. Research in aquaculture, apiculture, and non-timber forest sectors was limited, positioning these areas as opportunities for generating new knowledge, particularly through the analysis of local resources to enhance their market positioning while incorporating sustainability aspects.

## 1. Introduction

The value network approach involves the joint participation of various actors, including customers, suppliers, complementors, and competitors. These actors are linked through economic and non-economic relationships that contribute to value creation for their members and their territory [1]. Within a value network, these actors can collaborate more effectively. This facilitates the analysis of competitiveness across productive sectors and generates value for the system as a whole [2].

Unlike the linear and sequential nature of the traditional value chain, which focuses on endogenous factors that add value to a production system, the value network framework integrates both endogenous and exogenous factors within a dynamic system, where all actors interact in the same space and time. Due to its spatial, temporal, and adaptive components, this approach is frequently used to analyze the competitiveness of agro-industrial products [2].

While the value chain approach focuses on sequential, linear processes that add value to a product through a series of activities primarily within a firm, the value network approach extends this analysis to a broader ecosystem of actors (customers, suppliers, competitors, and complementors) linked through economic and non-economic relationships that collectively generate value for the territory [1]. Unlike value chains, value networks encompass non-linear, reciprocal interactions among agents operating simultaneously within the same spatial and temporal context [2].

The globalization of markets and the continuous search for new niches have triggered asymmetric competition in food production and consumption characterized by structural inequalities between large agri-food corporations with access to global distribution networks and local or smallholder producers with limited market reach and bargaining power [1]. In this context, competitiveness analysis has become essential for maintaining products in current markets. Accordingly, the value network approach has proven to be an efficient analytical tool, as it describes mutually beneficial relationships among the actors involved in production, market competition, and commercialization processes. This enhances the value of the products and/or services offered [3].

This framework has been applied in different agri-food systems such as wine [4] in Europe; maize [5] and coffee [6] in the Americas; rice [7] and sugarcane [8] in Asia; and beef production in Africa [9] and Oceania [10]. These applications have strengthened the position of these products in both regional and export markets. In Mexico, this methodology has consistently been used to analyze the competitiveness of agro-industrial value chains, including those of rice [11], maize [12], and Ataulfo mango [13], as well as aquaculture systems [14] and beverages such as beer [15], pulque [16], and mezcal [17].

Despite the growing number of publications using the value network approach to evaluate the competitiveness of agro-industrial products, knowledge gaps persist in productive sectors such as aquaculture, apiculture, and non-timber forest products. In these sectors, the approach could strengthen product trade [18] and contribute to the sustainability of production and commercialization systems [19].

Bibliometric techniques are effective tools for identifying research gaps on specific topics, because they generate indicators and mathematical models that characterize the development and evolution of scientific publications [20]. These techniques have been used to analyze the competitiveness of agro-industrial systems in general [21] and of specific products such as wine [22] and sorghum [23], contributing to improved market performance.

In this context, the objective of the present study was to analyze scientific publications addressing the competitiveness of food products within a value network framework using bibliometric techniques. This analysis aimed to determine the spatial and temporal distribution of the identified products and to detect research opportunities for future studies.

## 2. Materials and Methods

### 2.1. Data Source and Preparation

From January to March 2025, scientific articles applying the value network methodology to describe the competitiveness aspects of food-related products were collected. The search was conducted in the following indexing and abstracting databases: Scopus (Elsevier), Clarivate Analytics (Web of Science), SciELO, Redalyc, DOAJ, Latindex, PeerJ, and Conricyt. Publisher platforms (Elsevier, Springer, MDPI, and Frontiers) were used as complementary sources to retrieve full-text articles. Additionally, Google Scholar was employed as an open-access search engine to maximize coverage, with cross-verification against the indexed databases. The keywords used in the search were “value network” and “red de valor”, combined with descriptors related to “sustainable agricultural production” and “food security”.

Through a content analysis of each scientific article, those texts were included that applied the value network framework as their primary analytical approach and analyzed the competitiveness of a food product or productive system. Scientific articles were excluded where the value network concept was mentioned only as a secondary reference; the primary focus was supply chain logistics, traceability, or food safety without a competitiveness component; or the product analyzed did not belong to the food production sector. The following variables were extracted from the remaining studies: authors, year of publication, keywords, journal, title, citation count, language, institution, and country of origin of the first author, analyzed product or crop, productive sector, data collection techniques, and analytical methodologies.

**Figure 1:**
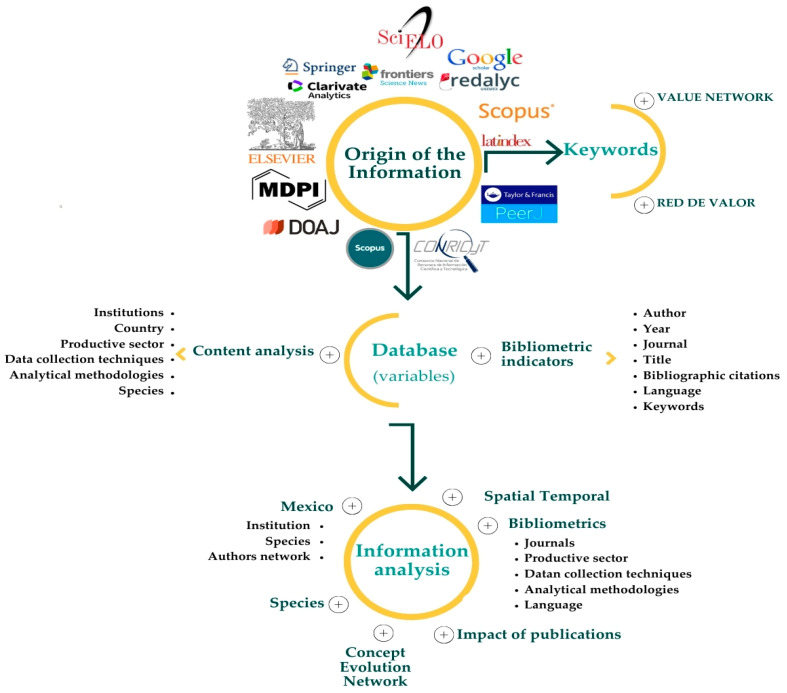
Flowchart of activities for the spatio-temporal analysis of scientific production, which applied the value network methodology to describe competitiveness aspects of food-value products.

All variables were recorded in a spreadsheet, preserving the original language of each article. Special characters such as “ñ”, accents, superscripts, subscripts, and trademark or copyright symbols (^®^, ©) were removed or standardized to facilitate analysis. This dataset served as the basis for spatial–temporal analyses, bibliometric indicators, publication impact assessment, co-occurrence networks of keywords and authors, and frequency analysis of the most relevant products or crops analyzed through the value network methodology. In addition, it enabled the identification of the main productive sectors, data collection techniques, and analytical methodologies used (Figure 1).

### 2.2. Spatial–Temporal Analysis

A temporal graph of scientific production was constructed using the publication year variable. To assess the annual frequency of scientific articles, an ordinary least squares (OLS) regression model was estimated to determine the publication frequency trend [24]. Additionally, the country of origin of the first author was georeferenced to identify where research using the value network methodology to describe competitiveness aspects of food-related products has been developed. The spatial representation was generated using the ArcGIS^®^ v10.8 geographic information system package [25].

### 2.3. Bibliometric Indicators

The following bibliometric indicators were generated to include journals with the highest publication frequency, publication language, productive sector (agricultural, livestock, perennial fruit, aquaculture, and apiculture), data collection techniques, and analytical methodologies.

### 2.4. Publication Impact

The number of publications and citations per country was determined using the variables number of citations, scientific articles, and the first author’s country of origin. This information was then used to create a graph illustrating the relationship between these two variables.

### 2.5. Concept Evolution Network

A temporal network of keywords was constructed using VOSviewer software v1.6.18 [26] to analyze the evolution of research that has employed the value network methodology to describe competitiveness aspects of food-related products. The keyword co-occurrence network was constructed using a minimum co-occurrence threshold of 3, with association strength as the normalization method. The network was visualized using the VOS layout algorithm. The cluster resolution was set to 1.0. The robustness of the clusters was assessed by varying the resolution parameter (±0.5).

### 2.6. Most Frequently Analyzed Food Species and Systems

A table was generated showing the frequency of crops (species) and systems (food products) by continent, in order to associate their importance with the dietary relevance of each region worldwide.

### 2.7. Perspectives in Mexico

Finally, to identify potential research areas in Mexico, we associated the frequency of crops and systems with the institution of origin of the first author. The spatial representation of this relationship was created using the ArcGIS^®^ geographic package [25]. In addition, a co-authorship network was built to identify key researchers who have contributed knowledge on the application of the value network methodology to assess the competitiveness of food-related products. This analysis was conducted using Gephi software v0.10.1 [27]. In Gephi, the ForceAtlas2 layout algorithm was used, with a modularity resolution of 1.0 (Louvain method). Only nodes with degree ≥ 2 were retained in the final visualization.

## 3. Results and Discussion

### 3.1. Spatial–Temporal Analysis

**Figure 2:**
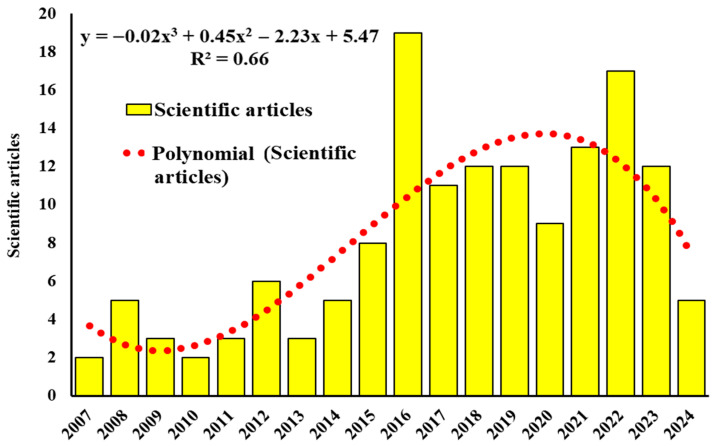
Temporal evolution of scientific production that applied the value network methodology to describe competitiveness aspects of food-value products from 2007 to 2024.

From 2007 to 2024, a total of 147 scientific articles applying the value network methodology to describe competitiveness aspects of food-related products were identified. The annual publication frequency (2007–2024) followed a significant quadratic trend (R^2^ = 0.66, *p* < 0.0001), characterized by an accelerating growth phase from 2007 to 2016 and a deceleration in the rate of growth from 2017 to 2024. The decreasing trend from 2017 to 2024 refers to the rate of annual increase (the first derivative of the quadratic function), not an absolute reduction in publication volume [24]; in fact, 61.90% of all articles (91 articles) were recorded in the 2017–2024 period (Figure 2). The concentration of 61.90% of all publications in the 2017–2024 period could suggest that the application of the value network methodology to food production sciences represents a developing research area, although this interpretation should be considered with caution, as the concentration of publications in recent years may also reflect factors such as the expansion of database coverage, changes in open-access policies, or increased interest in food security following global disruptions [20].

**Figure 3:**
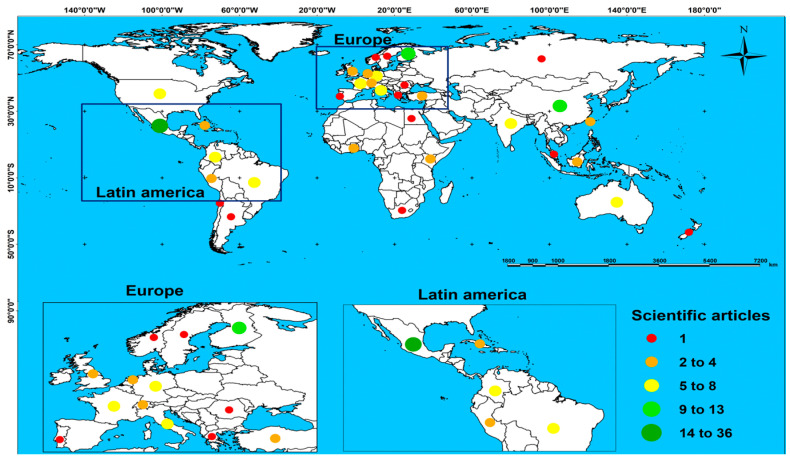
Spatial distribution of scientific production that applied the value network methodology to describe competitiveness aspects of food-value products from 2007 to 2024.

The 147 scientific articles originated from 33 countries (Figure 3). Nine countries accounted for 68.03% of all publications (100 scientific articles): Mexico (24.49%, 36), Finland (8.84%, 13), China (6.80%, 10), India (5.44%, 8), Italy (5.44%, 8), the United States (4.76%, 7), Germany (4.08%, 6), Brazil (4.08%, 6), and Colombia (4.08%, 6). Although the value network methodology initially emerged in the United States to assess competitiveness in manufacturing industry products, its application has become increasingly common in primary sector activities such as agriculture, livestock, fisheries, and forestry, especially in developing countries. One possible interpretation of the geographic concentration of publications in developing countries—particularly in Latin America—is that the value network methodology may be especially well-suited to analyzing economic actors in territorial contexts characterized by limited market formalization and products with a strong local identity [28,29]. This interpretation is consistent with the findings reported by Barrera-Rodríguez et al. [2] and Ricciotti [28], although additional empirical research would be needed to establish a causal relationship between territorial characteristics and the adoption of the methodology.

According to Barrera-Rodríguez et al. [2], the value network methodology has shown particular applicability in the analysis of local products whose commercialization is embedded in specific territorial contexts, where the identification of network actors and their relationships is facilitated by geographic proximity. Consequently, this methodology has primarily been applied to analyze the competitiveness of food-related products in countries with emerging economies, such as those in Latin America, where value network analyses have supported the market positioning of local products as substitutes for more globalized, and often imported, goods [29].

### 3.2. Bibliometric Indicators

The dispersion of 147 articles across 108 journals, with a mean of 1.34 articles per journal, suggests that the topic of competitiveness analysis of food-related products through the value network framework does not appear to be concentrated in any single specialized outlet. This pattern may reflect the interdisciplinary nature of the research area, which spans agricultural economics, food science, rural development, and management sciences, rather than the absence of potentially relevant specialized journals. Twelve journals accounted for 26.53% of the total publications (39 articles) (Table 1). These journals primarily focus on topics such as the bioeconomy, agriculture, and agribusiness. Those edited in English and indexed in the Journal Citation Reports [30] exhibited the highest citation counts. Among the 12 most productive journals, those edited in English and indexed in the Journal Citation Reports exhibited higher citation counts; this pattern suggests that publications in English in indexed journals tend to reach wider international audiences [20].

Approximately 77.79% of the publications were written in English (107 articles), 23.81% (35) in Spanish, and 3.40% (5) in Portuguese. According to Santillán-Fernández et al. [31], publishing in English increases the likelihood of dissemination within the scientific community, as it is the most widely used language among researchers. Of the 147 scientific articles, 104 (70.75%) analyzed the competitiveness of at least one food-related agricultural product, 13.61% (20) were focused on livestock products, 10.88% (16) on perennial fruit crops, 3.40% (5) on aquaculture, and 1.36% (2) on apiculture. Barrera-Rodríguez et al. [2] found that value network analyses are more frequent in agricultural systems due to the cyclical nature of crops. More importantly, because this sector contributes the most to food security in the regions where production occurs.

Among the instruments used for data collection, surveys (94 articles, 63.95% of the total) and semi-structured interviews (36, 24.49%) applied to the different actors within a value network were the most common. Qualitative techniques that capture social perceptions, such as participant observation, focus groups, field diaries, and the consultation of secondary sources, were reported in 17 publications (11.56%). Non-probabilistic snowball sampling was the most common strategy for implementing these instruments, due to the qualitative nature of the analyzed populations and the lack of a clearly defined sampling frame [32].

The information collected through these techniques enabled qualitative analyses such as value network characterization (focal firm, suppliers, customers, complementors, and competitors) in 25.85% of the studies (38 articles); innovation networks (nodes and linkages) in 34 articles (23.13%); SWOT analysis (Strengths, Opportunities, Weaknesses, and Threats) in 9 articles (6.12%); and problem tree analysis in 7 articles (4.76%). In addition, quantitative analyses such as cluster analysis (18, 12.24%), principal component analysis (14, 9.52%), and analysis of variance (16, 10.88%) were performed. However, despite the inherently spatial nature of value networks, there was no evidence using emerging technologies, such as remote sensing, geographic information systems, or satellite imagery, in these analyses. The incorporation of geostatistical models would therefore make it possible to introduce territorial components that have so far been scarcely explored.

### 3.3. Publication Impact

**Figure 4:**
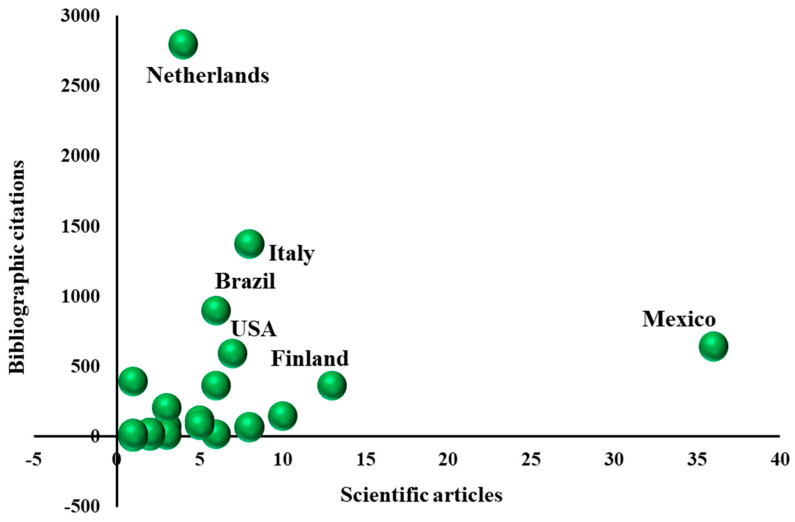
Impact of publications by country of origin of the first author, in studies that employed the value network methodology to describe competitiveness aspects of food-related products from 2007 to 2024.

When the impact of the publications was analyzed in terms of citation counts, Mexico and the Netherlands stood out (Figure 4); the former as the country with the most publications (36, 24.49% of the total), and the latter as the country with the most citations (2797, 33.57% of the total). Despite having fewer publications, Italy, Brazil, the United States, and Finland showed an impact equal to or greater than Mexico. Although the impact of Mexican publications was not the highest, their high productivity positioned Mexico as a pioneer in the application of the value network methodology to describe competitiveness aspects of food-related products [33].

The relatively low impact of Mexican publications can be explained, as suggested by Santillán-Fernández et al. [20], by the tendency of researchers to publish in institution-based journals edited in Spanish, which restricts constructive criticism and limits the visibility of their work. Since publications in English are more likely to be disseminated within the scientific community—because English is the most widely used language among researchers [31]—publishing in English is a key opportunity area for researchers in Mexico.

### 3.4. Concept Evolution Network

**Figure 5:**
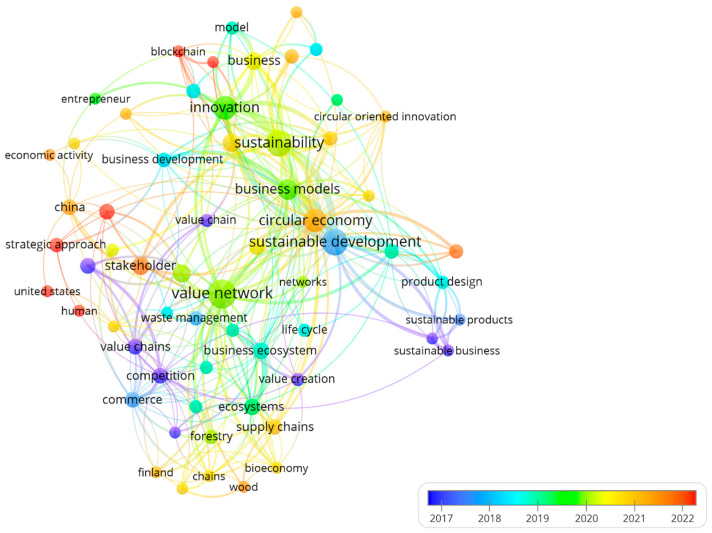
Co-occurrence network of keywords from scientific articles applying the value network methodology to food products (2007–2024), generated with VOSviewer. Node size is proportional to the frequency of occurrence of each keyword. Node color represents the thematic cluster to which each keyword belongs, based on the average publication year of the articles in which it appears: warm colors (yellow) indicate recently emerging terms (after 2018), while cool colors (blue/green) indicate terms from earlier stages (before 2015). Edge thickness reflects the co-occurrence intensity between pairs of keywords.

Until 2017 (Figure 5), the concept of a value network in food production sciences was considered similar to that of a value chain, since the value chain framework was a widely used methodology for analyzing agro-industrial competitiveness [2]. However, in recent years, the linear value chain model has gradually been replaced by the dynamic value network model. The latter incorporates the analysis of interactions among different production system actors, such as suppliers, customers, complementors, and competitors. This creates cooperative and competitive linkages among them and contributes to a better understanding and improvement of agro-industrial competitiveness [28].

Thus, the value network methodology has recurrently been employed in studies on the competitiveness of food-related products. Initially, these studies addressed the concept from a global, economic–capitalist perspective (before 2020). More recently (after 2020), these studies have incorporated notions of the circular economy, the bioeconomy, and sustainability in regional contexts (Figure 5). According to Schoneveld and Weng [34], the application of the value network methodology tends to be more effective in local agro-industrial systems that promote food security and sovereignty. It helps small-scale producers to develop strategies to remain in the market in the face of commercial monopolies.

### 3.5. Most Frequently Analyzed Food Species and Systems

Competitiveness analyses of food-related crops or production systems using the value network methodology focused mainly on products such as wine and bovine milk in Europe; maize, mango, rice, coffee, and lemon in the Americas; rice in Asia; and beef cattle in Africa and Oceania (Table 2). These crops and/or production systems were studied in regions where they are staple foods for the local population [35]. The Americas, Asia, and Europe concentrated 90.48% of the publications (133 articles). According to Hummer and Hancock [36], this is due to the large number of research centers located in these continents, which comprise the regions with a high population density.

Competitiveness analysis through the value network framework in agro-industrial systems proved to be the system that had the highest number of publications (31.97%, 47 articles). Government-driven promotion of sustainable food production systems has increased the number of competitiveness assessments aimed at contributing to food security and sovereignty [37]. In the context of food sovereignty, local resources play a major role. However, their markets are often limited by competition with globalized products. Therefore, analyzing their competitiveness across different market links (suppliers, customers, complementors, and competitors) helps generate strategies to maintain consumer preference [34].

**Table 2:** Main food-related crops and production systems by continent, documented in the scientific articles collected from 2007 to 2024 that employed the value network methodology to describe aspects of their competitiveness.

Continent	System		Scientific Article		Reference
	Common	Scientific	Number	%	
Africa (8, 5.44%)	Agroindustry		5	3.40	[34]
	Beef cattle	*Bos taurus* L.	2	1.36	[9]
	Tilapia	*Oreochromis niloticus* L.	1	0.68	[38]
America (62, 42.18%)	Agroindustry		8	5.44	[2]
	Maize	*Zea mayz* L.	5	3.40	[5]
	Mango	*Mangifera indica* L.	4	2.72	[13]
	Rice	*Oryza sativa* L.	4	2.72	[11]
	Coffee	*Coffea arabica* L.	3	2.04	[6]
	Lemon	*Citrus limon* L.	3	2.04	[39]
	Avocado	*Persea americana* Mill.	2	1.36	[40]
	Quinoa	*Chenopodium quinoa* Willd.	2	1.36	[41]
	Wheat	*Triticum aestivum* L.	2	1.36	[42]
	Sugarcane	*Saccharum officinarum* L.	2	1.36	[43]
	Beer		2	1.36	[15]
	Beef cattle	*Bos taurus* L.	3	2.04	[44]
	Cow milk	*Bos taurus* L.	3	2.04	[45]
	Goat milk	*Capra aegagrus hircus* L.	3	2.04	[46]
	Others		16	10.88	
Asia (23, 15.65%)	Agroindustry		6	4.08	[47]
	Rice	*Oryza sativa* L.	3	2.04	[7]
	Sugarcane	*Saccharum officinarum* L.	2	1.36	[8]
	Cow milk	*Bos taurus* L.	2	1.36	[48]
	Tea	*Camellia sinensis* L.	2	1.36	[49]
	Others		8	5.44	
Europe (48, 32.65%)	Agroindustry		25	17.01	[50]
	Wine	*Vitis vinifera* L.	4	2.72	[4]
	Cow milk	*Bos taurus* L.	4	2.72	[51]
	Aquaculture		3	2.04	[52]
	Others		12	8.16	
Oceania (6, 4.08%)	Agroindustry		3	2.04	[53]
	Beef cattle	*Bos taurus* L.	2	1.36	[10]
	Wine	*Vitis vinifera* L.	1	0.68	[54]
Total			147	100	

A limited application of the value network methodology was detected in aquaculture, apiculture, and non-timber forest products. According to Constantin et al. [18], this can be attributed to the fact that these sectors are characterized by high fragmentation and informality among producers, which complicates the identification and mapping of network actors; there is a marked absence of standardized national databases for these products, limiting the availability of secondary data required for competitiveness analysis; and the political economy of research funding in developing countries tends to prioritize staple crops (maize, rice, and wheat) over niche or emerging sectors.

### 3.6. Perspectives in Mexico

From 2007 to 2024, a total of 36 scientific articles employing the value network methodology to analyze the competitiveness of food-related products were published. The Universidad Autónoma Chapingo (UACh, 21 articles, 58.33%), Colegio de Postgraduados (ColPos, 5, 13.89%), and Instituto Nacional de Investigaciones Forestales, Agrícolas y Pecuarias (INIFAP, 2, 5.56%) collectively accounted for 77.78% of the total (28 articles) (Table 3). Traditionally, research published by the UACh (founded in 1854), the ColPos (founded in 1959), and the INIFAP (founded in 1985) has originated from initiatives aimed at advancing agricultural research and development. The most frequently analyzed crops were maize (4 articles, 11.11% of the total), mango (3, 8.31%), and lemons (2, 5.55%), all of which are staples of the Mexican diet [35].

**Figure 6:**
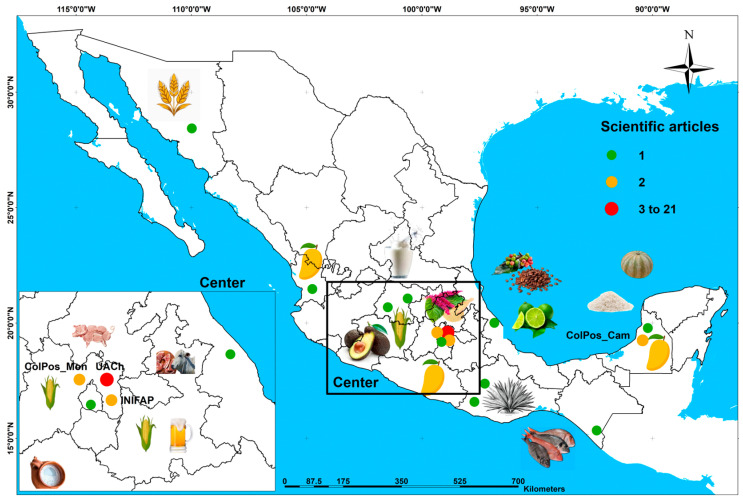
Spatial distribution of institutions in Mexico that produced scientific articles from 2007 to 2024, analyzing the competitiveness of food-related crops and systems using the value network methodology.

Figure 6 shows the spatial distribution of the institutions where these studies originated. It reveals that the main food-related crops and production systems analyzed through the value network methodology for competitiveness assessment were concentrated in central Mexico. Santillán-Fernández et al. [20,31] documented this centralization of research in Mexico and found that the territorial mismatch between production areas and research centers limits technology transfer, thereby affecting national agricultural competitiveness.

Mexico’s highest agricultural productivity is in the north, where intensive production systems are more competitive than the traditional extensive systems in central and southern Mexico [55]. Nevertheless, southern Mexico has the greatest population and agricultural crop diversity, positioning this region as a promising area for research development. However, to enhance the competitiveness of southern Mexico’s agricultural productivity, analyses using innovative approaches such as the value network methodology are needed to develop market positioning strategies at local, regional, national, and international levels [34]. This could also include exploring new market niches, such as edible forest species [31].

**Table 3:** Main food-related crops and production systems in Mexico, documented in the scientific articles collected from 2007 to 2024 that employed the value network methodology to describe aspects of their competitiveness.

Institution	System		Scientific Article		Reference
	Common	Scientific	Number	%	
UACh	Agroindustry		5	13.89	[2]
(21)	Maize	*Zea mayz* L.	3	8.31	[12]
	Lemon	*Citrus limon* L.	2	5.55	[39]
	Beef cattle	*Bos taurus* L.	2	5.55	[44]
	Goat milk	*Capra aegagrus hircus* L.	2	5.55	[46]
	Beer		2	5.55	[15]
	Avocado	*Persea americana* Mill.	1	2.78	[40]
	Rice	*Oryza sativa* L.	1	2.78	[11]
	Coffee	*Coffea arabica* L.	1	2.78	[6]
	Mango	*Mangifera indica* L.	1	2.78	[13]
	Wheat	*Triticum aestivum* L.	1	2.78	[42]
ColPos (5)					
Campeche (2)	Chihua	*Cucurbita argyrosperma*	1	2.78	[56]
	Mango	*Mangifera indica* L.	1	2.78	[57]
Montecillo (2)	Maize	*Zea mayz* L.	1	2.78	[5]
	Pork meat	*Sus scrofa domestica* L.	1	2.78	[58]
Veracruz (1)	Pulque	*Agave salmiana* L.	1	2.78	[16]
ECOSUR (2)					
Chiapas (1)	Agroindustry		1	2.78	[59]
Campeche (1)	Aquaculture		1	2.78	[14]
INIFAP (2)	Agroindustry		1	2.78	[60]
	Amaranth	*Amaranthus caudatus* L.	1	2.78	[61]
IPN (1)	Agroindustry		1	2.78	[62]
ITVO (1)	Cow milk	*Bos taurus* L.	1	2.78	[63]
UA_Nay (1)	Mango	*Mangifera indica* L.	1	2.78	[64]
UA_Gto (1)	Agroindustry		1	2.78	[65]
UNISON (1)	Agroindustry		1	2.78	[66]
NGO (1)	Mezcal	*Agave* spp.	1	2.78	[17]
Total			36	100	

UACh: Universidad Autónoma Chapingo; ColPos: Colegio de Postgraduados campus Montecillo, campus Campeche and campus Veracruz; ECOSUR: El Colegio de la Frontera Sur campus Chiapas and campus Campeche; IPN: Instituto Politécnico Nacional; ITVO: Instituto Tecnológico del Valle de Oaxaca; UA_Nay: Universidad Autónoma de Nayarit; UA_Gto: Universidad Autónoma de Guanajuato; UNISON: Universidad de Sonora; NGO: Non-Governmental Organization.

**Figure 7:**
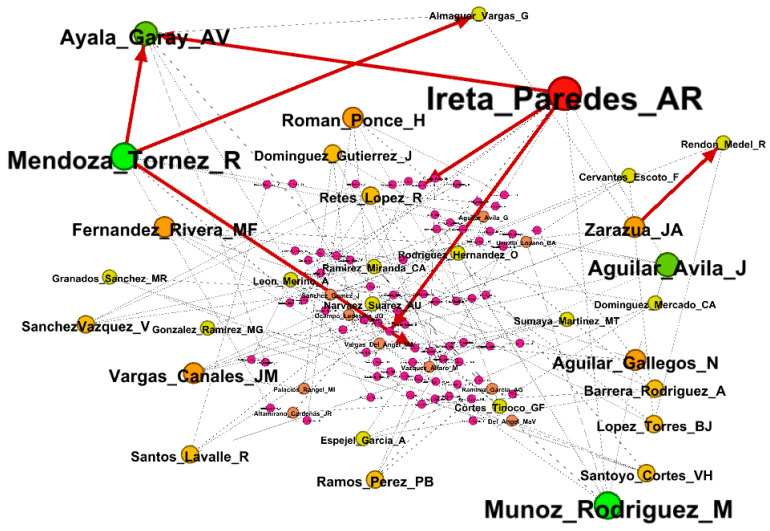
Author network in Mexico of scientific articles published from 2007 to 2024 that analyzed the competitiveness of food-related crops and systems using the value network methodology.

Figure 7 shows the author network. Researchers from Universidad Autónoma Chapingo (Ireta_Paredes_AR, Munoz_Rodriguez_M, Mendoza_Tornez_R, and Aguilar_Avila_J) and INIFAP (Ayala_Garay_AV) were identified as key contributors to the application of the value network methodology to determine the competitiveness of food-related crops and production systems in central Mexico. However, the analysis also revealed that authors primarily build research synergies mainly within their own institutions (114 nodes, 19 links, and a network density of 0.001), which limits constructive criticism and may affect the overall quality of the studies [20].

A co-authorship network density of 0.001 indicates very low collaboration intensity. Network density is defined as the ratio of observed connections to all possible connections among nodes; a value close to zero suggests that most authors operate within small, isolated collaborative clusters rather than forming a broad, integrated scientific community [67]; this fragmentation may reflect the disciplinary and geographic dispersion of researchers applying the value network methodology in different national contexts [20,31].

Therefore, conducting competitiveness analyses through the value network approach for local agricultural and forest food resources in southern Mexico represents an important opportunity to expand research efforts and foster collaborations with researchers from other institutions. This will ultimately contribute to national food security and sovereignty.

## 4. Conclusions

The analyzed scientific literature demonstrated the greatest growth from 2017 to 2024, with a focus on Latin American countries (maize, mango, rice, and coffee), Europe (wine and bovine milk), and Asia (rice and sugar). These studies focused on products from the region’s staple diets. There was limited research on aquaculture, apiculture, and forest products, which represents an opportunity to generate new knowledge, particularly through analyzing local resources to improve market positioning.

In aquaculture, the value network approach could map the complex interdependencies among producers, processors, distributors, and regulatory agencies in artisanal fishing systems; in apiculture, a value network analysis could capture the non-economic relationships among beekeepers, ecological service providers, and local communities that contribute to the sustainability of honey production systems; while for non-timber forest products, the value network approach could link biodiversity conservation actors with commercial value chains, supporting the development of payment-for-ecosystem-services schemes.

In Mexico, research was found to be spatially centralized and misaligned with production areas. It was led by researchers from the Universidad Autónoma Chapingo who studied products such as maize, rice, lemon, beef, and bovine milk. This presents an opportunity for researchers from northern and southern Mexico to analyze local resources within their areas of influence, which could contribute to enhanced competitiveness and food sovereignty.

Among the limitations of this study, it is acknowledged that the search, although comprehensive across open-access databases and indexed repositories, may have excluded articles published in restricted-access, high-impact specialized journals in the disciplines of agricultural economics and food policy. Therefore, it is recommended that future studies complement the search coverage by including restricted-access journals in these disciplines, in order to obtain a more complete bibliometric overview.

## Figures and Tables

**Table 1:** Bibliometric indicators of the main journals that published scientific articles employing the value network methodology to describe competitiveness aspects of food-related products from 2007 to 2024.

Journal	Country	Publisher	Topic	Language	WoS [30]	Scientific Articles	Citations
*JC_Production*	England	Elsevier	Bioeconomy	English	10 (Q1)	7	1940
*REMEXCA*	Mexico	INIFAP	Agriculture	Spanish	—	5	53
*AProd*	Mexico	ColPos	Agriculture	English	—	4	26
*RM_Agroneg*	Mexico	SMAA	Agribusiness	Spanish	—	4	23
*Sustainability*	Switzerland	MDPI	Sustainability	English	3.3 (Q1)	4	69
*Estudios Sociales*	Mexico	CIAD	Social Sciences	Spanish	—	3	110
*A_Systems*	England	Elsevier	Agriculture	English	6.1 (Q1)	2	103
*AH_Values*	The Netherlands	Springer	Bioeconomy	English	3.6 (Q1)	2	315
*AGROFOR_IJ*	Bos&Her	UE_Sarajevo	Agroeconomics	English	—	2	8
*C_Agroneg*	Brazil	UFRP	Agribusiness	Portuguese	—	2	6
*FP_Economics*	The Netherlands	Elsevier	Bioeconomy	English	3.8 (Q1)	2	57
*RMCP*	Mexico	INIFAP	Livestock Science	Spanish	0.7 (Q3)	2	12
Others (96)	—	—	—	—	—	108	5610
Total						147	8332

JC_Production: *Journal of Cleaner Production*; REMEXCA: *Revista Mexicana de Ciencias Agrícolas*; AProd: *AgroProductividad*; RM_Agroneg: *Revista Mexicana de Agronegocios*; A_Systems: *Agricultural Systems*; AH_Values: *Agriculture and human values*; AGROFOR_IJ: *AGROFOR International Journal*; C_Agroneg: *Custos e agronegocio*; FP_Enonomics: *Forest policy and Economics*; RMCP: *Revista Mexicana de ciencias pecuarias*; SMAA: *Sociedad Mexicana de Administración Agropecuaria A.C.*; CIAD: *Centro de Investigación en Alimentación y Desarrollo A.C.*; UE_Sarajevo: *University of East Sarajevo*; UFRP: *Universidad Federal Rural de Pernambuco*; INIFAP: *Instituto Nacional de Investigaciones Forestales, Agrícolas y Pecuarias*; ColPos: *Colegio de Postgraduados*; Bos&Her: Bosnia and Herzegovina.

## Data Availability

The data are available from the corresponding author upon reasonable request.
